# Role of isotropic lipid phase in the fusion of photosystem II membranes

**DOI:** 10.1007/s11120-024-01097-3

**Published:** 2024-04-25

**Authors:** Kinga Böde, Uroš Javornik, Ondřej Dlouhý, Ottó Zsíros, Avratanu Biswas, Ildikó Domonkos, Primož Šket, Václav Karlický, Bettina Ughy, Petar H. Lambrev, Vladimír Špunda, Janez Plavec, Győző Garab

**Affiliations:** 1 Institute of Plant Biology, HUN-REN Biological Research Centre, Szeged, Hungary; 2 Doctoral School of Biology, University of Szeged, Szeged, Hungary; 3 Department of Physics, Faculty of Science, University of Ostrava, Ostrava, Czech Republic; 4 Slovenian NMR Center, National Institute of Chemistry, Ljubljana, Slovenia; 5 Global Change Research Institute of the Czech Academy of Sciences, Brno, Czech Republic; 6 Faculty of Chemistry and Chemical Technology, University of Ljubljana, Ljubljana, Slovenia; 7 EN-FIST Center of Excellence, Ljubljana, Slovenia

**Keywords:** ^31^P-NMR spectroscopy; BBY membrane, Linear dichroism spectroscopy, Membrane fusion; non-bilayer lipids, Wheat germ lipase

## Abstract

It has been thoroughly documented, by using ^31^P-NMR spectroscopy, that plant thylakoid membranes (TMs), in addition to the bilayer (or lamellar, L) phase, contain at least two isotropic (I) lipid phases and an inverted hexagonal (H_II_) phase. However, our knowledge concerning the structural and functional roles of the non-bilayer phases is still rudimentary. The objective of the present study is to elucidate the origin of I phases which have been hypothesized to arise, in part, from the fusion of TMs (Garab et al. 2022 Progr Lipid Res 101,163). We take advantage of the selectivity of wheat germ lipase (WGL) in eliminating the I phases of TMs (Dlouhý et al. 2022 Cells 11: 2681), and the tendency of the so-called BBY particles, stacked photosystem II (PSII) enriched membrane pairs of 300–500 nm in diameter, to form large laterally fused sheets (Dunahay et al. 1984 BBA 764: 179). Our ^31^P-NMR spectroscopy data show that BBY membranes contain L and I phases. Similar to TMs, WGL selectively eliminated the I phases, which at the same time exerted no effect on the molecular organization and functional activity of PSII membranes. As revealed by sucrose-density centrifugation, magnetic linear dichroism spectroscopy and scanning electron microscopy, WGL disassembled the large laterally fused sheets. These data provide direct experimental evidence on the involvement of I phase(s) in the fusion of stacked PSII membrane pairs, and strongly suggest the role of non-bilayer lipids in the self-assembly of the TM system.

**Supplementary Information:**

The online version contains supplementary material available at 10.1007/s11120-024-01097-3.

## Introduction

In vascular plants, the light reactions of oxygenic photosynthesis occur in the multilamellar system of flattened membrane vesicles, the thylakoid membranes (TMs); they separate the inner and outer aqueous phases, the lumen and the stroma. TMs are differentiated into two well-discernible regions: the granum, consisting of stacked membranes, and the interconnecting unstacked stroma lamellae. Together they create a complex three-dimensional network of a continuous membrane enclosing a contiguous lumenal aqueous phase (Mustárdy and Garab 2003). The extensive vesicular network of TMs is characterized by a high degree of connectedness, attributed to multiple connections and membrane fusions, enhancing molecular trafficking both within the membranes and the lumenal space (Bussi et al. 2019). The grana and stroma regions display lateral heterogeneity regarding their protein compositions (Dekker and Boekema 2005). Photosystem II (PSII) and light-harvesting complex II (LHCII) are located predominantly in the stacked regions; photosystem I (PSI) and LHCI, and the ATP synthase are found in the stroma membranes; the cytochrome b_6_f complexes are evenly distributed.

The two photosystems and their associated LHCs form highly organized supercomplexes, which warrants efficient excitation energy transfer between the pigment-protein complexes and the funneling of excitons to the photochemical reaction centers (RCs). The first steps of the conversion of light energy to chemical energy occur in the RCs. These primary events are followed by vectorial electron and proton transport processes, which generate a transmembrane ΔpH (acidification of the lumen by 2–3 pH units) and an electric potential gradient (ΔΨ, of approximately 10^5^ V cm^−1^); ΔpH and ΔΨ are components of Δµ_H_^+^, the electrochemical potential gradient for protons, also known as the proton-motive force (pmf), which under steady-state conditions is almost entirely consist of ΔpH (Blankenship 2021). Generation and utilization of Δµ_H_^+^ for ATP synthesis according to the chemiosmotic mechanism (Mitchell 1966) is warranted by the organization of the bulk lipid molecules into bilayer structures, which prevents the penetration of water, most water-soluble molecules, and ions through the membrane (Singer and Nicolson 1972).

The major lipids of TMs are the mono- and di-galactosyldiacylglycerol (MGDG and DGDG), which account for about 50% and 30% of the total lipid content, respectively. TMs also contain sulfoquinovosyldiacylglycerol (SQDG) (~ 5–12%) and phosphatidylglycerol (PG) (~ 5–12%) (Douce and Joyard 1996; Harwood 1998; Boudière et al. 2014). While, as pointed out above, the bilayer organization of the bulk lipids plays key role in the build-up and utilization of pmf, it is well known that MGDG is a non-bilayer or non-lamella forming lipid (in this paper, we refer to these lipids as non-bilayer lipids) (Graham Shipley et al. 1973). Non-bilayer lipids, because of their conical molecular shapes (Israelachvili et al. 1980) prefer to adopt ordered mesophases or non-lamellar/non-bilayer structures with structural arrangement spanning from 2 to 3 nm up to several hundreds of nanometers (Conn and Seddon 2014). These lipids tend to assemble into inverted hexagonal (H_II_), isotropic (I) and cubic phases (Williams 1998; Epand 1998; Gounaris et al. 1986). This holds true also for lipid mixtures with high non-bilayer propensity.

Non-bilayer lipids are present probably in all biological membranes, in which, nevertheless, the bulk lipids are thought to be arranged as bilayers (van den Brink-van der Laan et al. 2004; Epand 1998). To explain the structural and functional roles of non-bilayer lipids, membrane models have been proposed that “challenge the ‘standard’ model (the fluid mosaic model) found in biochemistry texts” (Brown 2012). The Lateral Pressure Model (LPM) proposes that the non-bilayer lipids’ functional role lies in exerting lateral pressure in the hydrophobic region of the bilayer membrane, hence “keeping the [proteins] in a functional state, whereas in the absence of such force the proteins become less efficient or nonfunctional” (de Kruijff 1997). According to the Flexible Surface Model (FSM) “the non-lamellar-forming tendency of the membrane lipids modulates the protein energetics” via variations in the curvature elastic energy (Brown 2012). These models consider non-bilayer lipids as integral parts of the bilayer phase, and non-bilayer phases to occur within the bilayer only locally and transiently (van den Brink-van der Laan et al. 2004). In other terms, neither LPM nor FSM consider persisting non-bilayer lipid phases and occurrence of lipids outside the bilayer membrane. In contrast, the Dynamic Exchange Model (DEM) – proposed for energy converting membranes, in which the major lipids are non-lamella-forming lipid species – presumes the coexistence of bilayer and non-bilayer lipid phases in a dynamic equilibrium (Garab et al. 2000, 2016). DEM is based on the Janus face of non-bilayer lipids. It has been shown that, on the one hand, isolated MGDG is forced into a bilayer structure upon the addition of isolated LHCII, forming extended LHCII:MGDG lamellar aggregates (Simidjiev et al. 2000). On the other hand, lipid mixtures with high non-bilayer propensity are known to be capable of segregating from membranes that contain large protein-free membrane patches exposed to water (Williams 1998; Seddon and Templer 1995). By this means, non-bilayer lipids have been suggested to play a self-regulatory role in governing the homeostasis of TMs and the inner mitochondrial membranes (IMMs), controlling the high (~ 75:25) protein-to-lipid ratio in these basic energy-converting biological membranes (see also (Garab et al. 2000, 2022)).

DEM gained support from experiments, using ^31^P-NMR spectroscopy, which provided evidence for the coexistence of bilayer and non-bilayer phases in isolated intact spinach TMs (Krumova et al. 2008a). ^31^P-NMR spectroscopy is widely used to characterize the phase behavior of phospholipids in both natural and artificial membranes (Cullis and de Kruijff 1979; Schiller et al. 2007; Watts 2013). In TMs, it monitors the motion of bulk PG molecules; PG can serve as a sensitive indicator of the phase behavior of TM lipids (Harańczyk et al. 1985, 1995) because it shows no lateral heterogeneity in the bulk phase (Duchene and Siegenthaler 2000; van Eerden et al. 2015). Experiments on a large number of fully functional TMs, isolated from different higher plant leaves, have provided evidence for the presence of three different non-bilayer phases: beside the bilayer (or lamellar, L) phase, an inverted hexagonal (H_II_) phase and at least two isotropic phases (Garab et al. 2017, 2022). Concerning the distribution of lipids in different phases it has been estimated that only about 40% of the bulk lipids are present in the bilayer, and about the same amount is located in the H_II_ phase; the I phases contain the remaining lipids, about 20% (Dlouhý et al. 2021a). It has also been shown that isolated grana and stroma lamellae display very similar lipid polymorphism as the TMs; thus, ruling out the role of protein composition in determining the phase behavior of bulk TM lipids (Dlouhý et al. 2021a). Steady-state and time-resolved fluorescence spectroscopy data, using TMs stained with the lipophilic fluorescent probe Merocyanine-540, are in harmony with the polymorphic phase behavior detected by ^31^P-NMR spectroscopy (Krumova et al. 2008b; Garab et al. 2017). The lipid phase behavior of TMs has been shown to depend strongly on the pH and ionic strength of the medium as well as on the temperature (Kotakis et al. 2018; Ughy et al. 2019), which underpins the importance of lipid polymorphism in the energization of TMs. The lipid phase behavior of TMs is also modulated by the saturation of double bonds of fatty acyl constituents of the membrane lipids (Garab et al. 2017); thermotropic behavior of the non-bilayer and bilayer glycolipids of the green alga *Ulva lactuca* has been proposed to contribute to maintenance of the highly dynamic structure of TMs (Kostetsky et al. 2018). Reversible low-pH and elevated-temperature induced enhancements of one of the isotropic phases of TMs have been correlated with the photoprotective activity of the water-soluble lumenal enzyme, violaxanthin de-epoxidase (VDE) (Dlouhý et al. 2020) – in good agreement with reports on the role of non-bilayer lipid phase in regulating VDE activity in model membrane systems (Latowski et al. 2002, 2004; Goss and Latowski 2020). The other I phase of TMs was hypothesized to be associated with membrane fusions and junctions (Garab et al. 2017; Dlouhý et al. 2021b).

Comprehending the process of biological membrane fusion is of utmost importance considering its key role in numerous cellular processes such as cellular communication, cell growth, vesicle trafficking, organelle biogenesis and membrane dynamics (Stefan et al. 2017; Rast et al. 2015). Despite intense research, our current understanding of the molecular and physical mechanism of membrane fusions is far from being complete (Brukman et al. 2019). In general, the process of biological fusion is reliant upon the lipid composition of membranes, which modulate various factors such as membrane dynamics, hydration forces, and interactions between lipids and proteins (Meher and Chakraborty 2019; Pabst et al. 2014). Research in the field of protein-mediated fusion events primarily revolves around viral fusogenic proteins, e.g., HIV gp41 protein, influenza hemagglutinin. Additionally, SNARE proteins have been identified as key players in facilitating membrane fusions (Risselada and Grubmüller 2012). In TMs, the 30 kDa inner membrane-associated protein (IM30) also known as the vesicle-inducing protein in plastids 1 (vipp1), with roles in TM biogenesis and viability (Gao and Xu 2009), and Fzl, involved in photoprotection, have been demonstrated to participate in membrane fusion processes (Siebenaller et al. 2019; Findinier et al. 2019). However, it has also been clarified that membrane fusion can proceed in the absence of dedicated proteins (Chernomordik and Kozlov 2003). Non-bilayer lipids, with preference for non-lamellar structures with negative curvature, play a significant role in inducing and stabilizing fusion events that proceed via a highly curved, crucial intermediate structure called hemifusion (Chernomordik and Kozlov 2008; Marrink et al. 2009; Akimov et al. 2020; Joardar et al. 2022; Yaghmur et al. 2014). The initial hemifusion connection, termed the fusion stalk, also serves as the primary intermediate during the transition from a lamellar phase to an inverted hexagonal (H_II_) phase (Kozlov et al. 1989; Siegel and Epand 1997; van Eerden et al. 2015). The fact that both protein-mediated fusion and pure lipidic fusion culminate in the formation of hemifusion/stalk structures, it is highly plausible that the fundamental mechanism behind this process is predominantly governed by the physics of lipid interactions (Chernomordik and Kozlov 2003).

The main objective of this study is to test the hypothesis on the role of isotropic lipid phase in the fusion of TMs. It is well established that network formation and structural dynamics of TMs depend largely on their fusion and branching capabilities (Bussi et al. 2019; Garab et al. 2022). Further, isolated granum and stroma subchloroplast particles have also been shown to assemble into large vesicular structures (Dlouhý et al. 2021b), which strongly indicates the occurrence of spontaneous fusion of TMs. To provide direct experimental evidence for the involvement of I phase(s) in membrane fusion, here we use PSII-enriched membrane particles (BBY membranes), “highly purified grana membrane fractions composed of paired, appressed membrane fragments,” which are cca. 300–500 nm in diameter, and tend to form “large, flattened sheet-like appearance of the membranes, caused by the lateral fusion of membrane fragments” (Dunahay et al. 1984). BBY particles are widely used units of TM to study the structure and function of PSII. Detergent (digitonin and Triton X-100) fractionation of whole TMs has been widely employed to separate the stacked grana membranes from the unstacked stroma lamellae (Dunahay et al. 1984); Triton X-100 has been shown to be superior (compared to digitonin) in yielding highly active PSII membranes (Yamamoto et al. 2011).

BBY particles are here shown to display L and I phases albeit, because of their diminished lipid content (Haferkamp and Kirchhoff 2008), with lower intensities than in TMs. By using sucrose-density centrifugation, magnetic linear dichroism spectroscopy and scanning electron microscopy, we show that wheat germ lipase (WGL), a substrate non-specific/promiscuous lipid hydrolase enzyme (Kublicki et al. 2021), which has earlier been shown to selectively eliminate the I phases of TMs (Dlouhý et al. 2022), disassemble the large sheets of BBY to smaller entities. Hence, these data provide direct experimental evidence on the involvement of I phase in the fusion of PSII membranes, and strongly suggest the role of non-bilayer lipids in the self-assembly of the TM system.

## Materials and methods

### BBY isolation

For the isolation of PSII-enriched thylakoid membrane particles (BBY) fresh spinach leaves were purchased from the local market. In the isolation procedure we followed the step-by-step protocol of Yamamoto and coworkers (Yamamoto et al. 2011), which is based on the use of the nonionic detergent Triton X-100 for a 10 min period of time to separate physically the granum and stroma TMs from each other. BBY particles, obtained after differential centrifugation are composed of stacked PSII membrane pairs of high oxygen-evolving activity. The estimated residual detergent concentration does not exceed 0.035%, which – as supported by all literature data and our circular dichroism (CD) spectroscopy and fast chlorophyll-a (Chl-a) fluorescence transient data (see below, and the Supplementary Material) – does not perturb the structure and function of these membranes. Indeed, as pointed out by Schiller and Dau (Schiller and Dau 2000), these widely used particles that are „obtained by a partial detergent solubilization method […are thylakoid] membrane patches containing intact and highly active PS II”. (CD spectroscopy and fast Chl-a fluorescence transients are widely used highly sensitive non-invasive techniques. CD spectroscopy is capable of detecting minor alterations in the short-range, excitonic interactions in the pigment system (Garab and van Amerongen 2009); fast Chl-a fluorescence transients are used to monitor the activity of PSII (Govindjee and Papageorgiou 2004; Garab et al. 2023).

Freshly isolated BBY particles were either kept on ice and used for measurements on the same day, or were stored for several days or weeks at -80 °C until use; in some cases, the freshly prepared samples were exposed to freeze-and-thaw cycles – all these samples yielded very similar results in all experiments. The Chl (a + b) content of the sample was determined according to (Porra et al. 1989).

### Sonication of BBY particles

Sonication of BBY was carried out on ice with a Vibracell Model VCX 750 (Sonics and Materials, Danbury, CT, USA) equipped with a 3 mm diameter probe tip for 3 × 15 s, with 1-min resting intervals. The ultrasonic exposure had an intensity of 20% duty pulse.

### Treatments with wheat germ lipase

Isolated BBY membranes were treated with wheat germ lipase (WGL) – a substrate nonspecific general tri-, di-, and monoglyceride hydrolase/lipase (Kublicki et al. 2021). WGL was purchased from Sigma-Aldrich (Burlington, MA, USA) and was applied in the range of 5–50 U mL^−1^ activity; the treatments and incubations were performed at 5 °C. Earlier, using thin layer chromatography, we have verified that WGL digests the main TM lipid species, MGDG (Dlouhý et al. 2022) – these data are now shown in SFig. 1.

### ^31^P–NMR spectroscopy measurements

^31^P-NMR spectrometry was performed as described in (Dlouhý et al. 2021a). Spectra were obtained on an Avance Neo 600 MHz NMR spectrometer (Bruker, Billerica, MA, USA) equipped with a BBFO SmartProbe that was tuned to the phosphorus frequency. Approximately 700 µL of the sample was loaded into 5 mm diameter NMR tubes.

Due to the high density of the samples, leading to the random coagulation of TMs and BBY particles, magnetic orientation of the sample did not occur. This was previously demonstrated by stirring the TM suspension, which exerted no discernible effect on the ^31^P-NMR spectra (Krumova et al. 2008a). In unreported experiments, we verified that no magnetic alignment of BBY membranes occur already at 2.5 mg mL^−1^ Chl content of the suspension, which is about four times less than used in ^31^P-NMR spectroscopy (Dlouhý et al. 2022); this is evidently due to the formation of random aggregates of the particles which hinders their magnetic orientability.

For spectra acquisition, 40° RF pulses with an inter-pulse time of 0.5 s were applied without ^1^H-decoupling, as in earlier experiments (Krumova et al. 2008a). Indeed, experiments employing inverse gated decoupling using a waltz16 composite pulse scheme at 3.5 kHz field strength, we verified, that the recorded spectra were indistinguishable in the presence and in the absence of ^1^H-decoupling (data not shown). The same held true when, instead of 0.5 s, four times longer (2 s) inter-pulse time was employed. The corresponding spectra are displayed in the Supplementary Material (SFig. 2). We also verified that the spectral baseline, under the applied experimental setup was of good quality, confirmed through the recording of a spectrum of the probe background – eliminating the potential influence of baseline distortion on signal fitting. A pre-scan delay of 6.5 µs was used in the experiments. This could suppress signal modulated by dipolar coupling on the order of 10 kHz and more, but such couplings exceed what is expected in biological membrane systems. It is important to note, however, that Hahn echo technique was not used in our experiments, since it acts as a T2 filter, potentially altering the apparent signal ratios from species with different relaxation times. As an external chemical-shift reference 85% solution of H_3_PO_4_ in water was used.

In the saturation transfer (ST) experiments, we employed RF pulses with low power at the designated frequency for a duration of 0.3 s, followed by 40° pulses with an acquisition time of 0.2 s and a repetition time of 0.5 s. The intensity of the pre-saturation pulse was adjusted based on the intensity of the saturated peak. For the pre-saturation RF pulses, the field strengths were set to 80 Hz for lamellar and 40 Hz for isotropic phases.

^31^P-NMR spectra were normalized with respect to both the Chl contents and the number of scans, then the normalized spectra of different samples were averaged to improve the signal-to-noise ratio. For ^31^P-NMR data processing, TopSpin software (Bruker, Billerica, MA, USA) was used; the mathematical deconvolution of spectra was carried out using DMfit software (Dominique Massiot, Orléans, France) (Massiot et al. 2002), which yielded spectral shapes characteristic of different lipid phases (Cullis and de Kruijff 1979; Watts 2013). The figures were plotted using MATLAB R2020b (MathWorks, Inc., Portola Valley, CA, USA) with an implemented Spectr-O-Matic toolbox for the analysis of spectroscopic data (Dr. Petar H. Lambrev, Szeged, Hungary).

### Sucrose density gradient (SDG)

Untreated, enzyme treated and sonicated samples were loaded onto SDG (0.5–1.5 M sucrose) in a buffer containing 50 mM Tricine, 5 mM MgCl_2_, 5 mM KCl, pH 7.5) and centrifuged at 4 °C in a swinging-bucket rotor for 10 min at 3,000 × g. After centrifugation the tubes were photographed and converted into 8-bit greyscale image using ImageJ (Rasband, W.S., ImageJ, U. S. National Institutes of Health, Bethesda, Maryland, USA). Ten equally sized regions were selected along the tube as regions of interest (ROI). In each ROI, we determined the mean pixel intensity (PI) value, which fell between 0 and 255 (= PI_max_). By this means, PI_max_ - PI is characteristic of the optical density of the selected volume.

### Magnetic linear dichroism (MLD) spectroscopy

Linear dichroism was recorded from untreated and WGL-treated samples using a JASCO J-815 spectropolarimeter (Jasco, Tokyo, Japan) in the wavelength range from 400 to 750 nm, at 100 nm min^−1^ scan speed. Measurements were carried out at room temperature, with a bandwidth of 2 nm and data pitch of 1.0 nm. The Chl concentration of the samples was adjusted to 30 µg mL^−1^ and measured in a quartz cuvette with 1 cm optical path length. The enzymatic treatments of different enzyme concentrations started immediately before placing the cuvettes into the magnetic sample holder providing a magnetic field strength of 0.7 T (Kiss et al. 1986). The external magnetic field aligned the membranes with their planes perpendicular to the field vector, thus offering edge-aligned position of the membranes (Garab and van Amerongen 2009).

### Scanning electron microscopy (SEM)

Control and WGL-treated membrane fractions were fixed in 2.5% glutaraldehyde, oriented parallel to the surface by external magnetic field and settled on poly-L-lysine-coated polycarbonate filter for 1 h. After post-fixation in 1% OsO_4_ for 50 min, the samples were dehydrated in aqueous solutions of increasing ethanol concentrations, critical point dried, covered with 5 nm gold by a Quorum Q150TES (Quorum Technologies, Lewes, UK) sputter, and observed in a JEOL JSM-7100 F/LV scanning electron microscope (JEOL, Tokyo, Japan).

### Fast Chl-a fluorescence transient measurements

Fast Chl-a fluorescence transients of BBY particles at Chl concentrations of 20 µg mL^−1^ were measured using a Handy-PEA Chl-a fluorimeter (Hansatech Instruments Ltd., Pentney, United Kingdom). Samples were dark adapted for 10 min and illuminated with red light at a photon flux density of 1,500 µmol photons m^−2^ s^−1^ for 3 s. The F_v_/F_m_ ratio, characterizing the photochemical activity and structural dynamics of PSII (Sipka et al. 2021) was calculated as (F_m_-F_o_)/F_m_, where F_m_ and F_o_ are maximal and minimal fluorescence levels, respectively.

### Circular dichroism spectroscopy

Circular dichroism spectra of untreated and WGL-treated samples were recorded using a JASCO J-815 spectropolarimeter (Jasco, Tokyo, Japan) between 400 and 750 nm at 100 nm min^−1^ scan speed. Measurements were carried out at room temperature, with a bandwidth of 2 nm and data pitch of 1.0 nm. The Chl concentration of the samples was adjusted to 30 µg mL^−1^ and measured in a cuvette with 1 cm optical path length. The CD spectra were normalized to the absorbance maxima at around 680 nm, with a reference wavelength at 750 nm. The enzymatic treatments of different concentrations started immediately before placing the cuvettes into the sample holder.

### Fourier-transform infrared (FTIR) spectroscopy

For FTIR measurements, 100 µL TM or BBY suspension of 4–5 mg Chl mL^−1^ was diluted in 1 mL D_2_O-based PBS solution and centrifuged at 10,000 × g for 2 min (PBS, phos-phate-buffered saline). After discarding the supernatant, the pellet was resuspended in D_2_O-based PBS solution again. These steps were repeated three times, for complete H_2_O to D_2_O exchange. The final pellet was layered between CaF_2_ windows, separated by an aluminum spacer, and placed in a Bruker Vertex70 FTIR spectrometer using a temperature-regulated shuttle sample holder. Spectra were recorded between 4,000 and 900 cm^−1^, 512 interferograms were collected for each spectrum, the spectral resolution was 2 cm^−1^. The infrared absorption spectrum of the samples was calculated from the background and sample of single beam spectra with Opus software of Bruker.

For the analysis of the structural properties of the membrane, the ester C = O plus the Amide I region, which in this paper will be referred to as ‘Ester + Amide I’ region was used between 1,800 and 1,595 cm^−1^. In the ‘Ester + Amide I’ region, to obtain the relative intensities of the C = O and the Amide I bands, Skew-Gaussian fit was applied (Zucchelli et al. 1990). In the selected region, 3rd order polynomial was fitted and subtracted as baseline. All data analyses were performed by using built-in MATLAB functions.

## Results and discussion

### Lipid polymorphism of BBY membranes determined by ^31^P-NMR spectroscopy

#### Distribution of bulk lipid molecules among the L and I phases

It has been thoroughly demonstrated that ^31^P-NMR spectroscopy provides information on the phase behavior of bulk lipid molecules in TMs and granum and stroma subchloroplast particles (Krumova et al. 2008a; Garab et al. 2017; Dlouhý et al. 2021a). Here we applied this technique for BBY particles, highly active oxygen-evolving stacked PSII membrane pairs that are laterally fused to form large sheets.

**Fig. 1:**
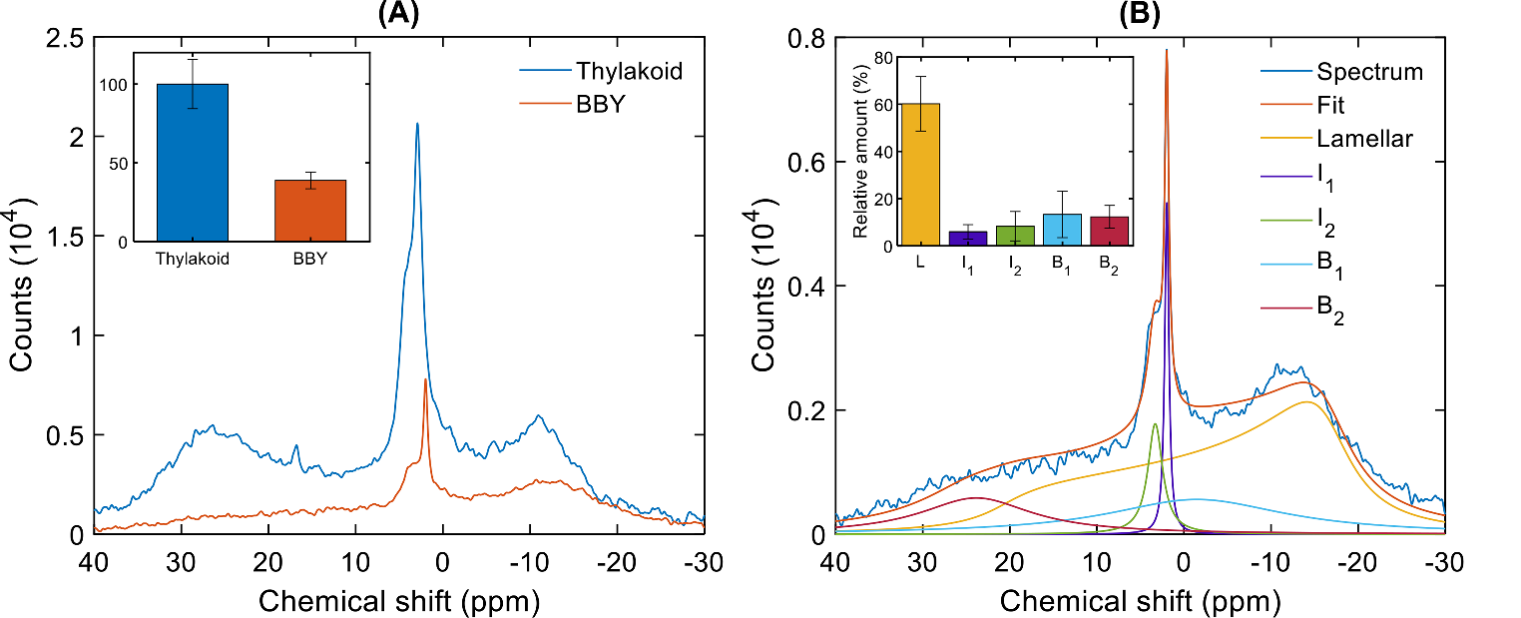
^31^P-NMR spectra of freshly isolated BBY particles and TM membranes (**A**) and mathematical deconvolution of the spectrum of BBY (**B**). Panel **A**: Average spectra from 12 (BBY) and 10 (TM) biological replicates. The spectra are normalized to identical Chl concentration (10 mg mL^-1^) and the number of scans (1600). Inset, areas under the curves; for easier comparison, the integrated area of TM is taken as 100. Panel **B**: The averaged spectrum of BBY and deconvoluted spectral components and their sum. Inset, relative contributions of the different spectral components, mean values ± SD

As it can be seen in Fig. 1A, the ^31^P-NMR spectrum of freshly isolated BBY membranes shared similarities with the spectrum of TM, albeit with some well-marked differences. When compared to TM, the overall signal intensity in the spectra of BBY was considerably lower, 38.8 ± 5.3% (*n* = 10) (Fig. 1A inset) of that in TMs isolated and measured under similar conditions. These data are in good agreement with earlier reports demonstrating that the lipid content of BBY particles, prepared by using detergent, is significantly lower than that of granum TMs obtained after mechanical fragmentation (Haferkamp and Kirchhoff 2008) and of intact TMs (Kirchhoff et al. 2002). The reduced lipid content of BBY compared to TM was also confirmed by our FTIR spectroscopy measurements (SFig. 3).

Another marked difference between the lipid polymorphism of BBY particles and TMs was the absence of the H_II_ phase, i.e. the lack of the broad asymmetric band around 25 ppm extending toward the high-field side (Garab et al. 2022). Mild trypsin treatment - which eliminates the H_II_ phase in TMs (Dlouhý et al. 2022) – exerted no effect on the ^31^P-NMR spectrum of BBY particles (data not shown). The H_II_ phase in TMs has been suggested to originate from lipids encapsulating stromal side proteins or polypeptides (Dlouhý et al. 2022). BBY particles, composed of stacked PSII membranes, lack stroma exposed regions – which explains the absence of H_II_ phase.

Similar to TMs, BBY particles displayed the ^31^P-NMR spectral signature of L phase, a broad band peaking at around  -10 ppm and extending towards the low-field side of the spectrum. In addition, the spectra also contained sharp isotropic peaks between 0 and 5 ppm, resembling those found in TMs. Indeed, mathematical deconvolution of the averaged (*n* = 12) ^31^P-NMR spectrum of BBY resolved L phase and two sharp isotropic peaks (I_1_ and I_2_) centered at approximately 2 and 3.5 ppm, respectively (Fig. 1B). The L phase constituted 60.2 ± 11.6% of the total intensity as derived from the deconvolution of spectra on 12 preparations. The I_1_ and I_2_ phases were present with intensities of 5.9 ± 3.0% and 8.3 ± 6.2%, respectively. It is interesting to note that the relative contribution of the I phases in BBY (~ 14%) does not differ significantly from that in TMs (~ 16%) (Supplementary Material SFig. 4).

In BBY, saturation transfer experiments and the mathematical deconvolution indicated the presence of two further components with broad symmetric bands (B_1_ and B_2_) located approximately at 0 and 20 ppm, with intensities of 13.3 ± 9.6% and 12.3 ± 4.8%, respectively, suggesting a more complex band structure than anticipated. It is to be noted that the signal-to-noise ratio, especially that of B_2_, may not allow us to make firm statement about its spectral distribution. Nonetheless, their characterization might be worthwhile, as they appear to indicate minor alterations in the lipid phase behavior of BBY particles compared to intact TMs. (Spectra recorded in saturation transfer experiments are displayed in the Supplementary Material, SFig. 5.)

Regarding the I phases, “isotropic averaging spectral components” have earlier been observed in a variety of biological and model membranes (de Kruijff et al. 1980). Hence, their appearance in TMs and BBY membranes, with high abundance of MGDG, is not really unexpected. As we reported earlier, the peak positions of the isotropic phases in TMs vary in a relatively broad interval upon varying the temperature and pH (Dlouhý et al. 2020) – these largely reversible changes suggest that the peak positions and relative amplitudes of I phases depend on the physico-chemical environment of lipids. Heterogeneity of the lipid environment in TMs is also indicated by time-resolved fluorescence spectroscopy, using the lipophilic fluorescence dye Merocyanine-540, which is sensitive to the local dielectric constant (Krumova et al. 2008b; Garab et al. 2017; Kotakis et al. 2018).

The I phases, which are present in all our intact TMs and subchloroplast membrane particles, including BBY, evidently originate from small and fast-tumbling objects. As revealed by our systematic studies in the past years, the presence of these objects are hallmarks of all functional TMs and subchloroplast particles. ^31^P-NMR spectra with similar features might indicate the presence of lipid droplets. However¸ as concerns “the plastoglobule lipid droplet, a dynamic sub-compartment of plant chloroplast” (Shivaiah et al. 2022), we have earlier ruled out their contribution to the observed polymorphism of TMs (Dlouhý et al. 2022). Other types of lipid droplets might accumulate in chloroplasts, e.g. of high-light exposed *Chlamydomonas* (Goold et al. 2016). Although in TMs, the occurrence of lipid droplets cannot be ruled out, it is highly unlikely that they would significantly contribute to TMs’ lipid polymorphisms, which appear to be very robust, observed in all our TM, including intact TMs isolated from young, mature and senescing pea leaves (unpublished data). Further, we are not aware of literature data reporting the presence of lipid droplets in BBY, which, compared to TMs, are deficient in lipids.

Concerning the emergence of the broad bands, B_1_ and B_2_, we point out that no similar bands are present either in intact TMs or in granum and stroma membrane preparations (Dlouhý et al. 2021a; Garab et al. 2022). We ascribe them as unidentified components. They might originate from lipid molecules partially detached from the lipid shell of supercomplexes upon the effect of Triton X-100 applied during the isolation procedure. These shell or annular lipids are normally ^31^P-NMR silent in TMs. However, they might be at least partially solubilized by Triton X-100, in a manner similar to the effect of this detergent during LHCII preparation (Simidjiev et al. 1997). Clarifying the exact origin of these bands is outside the scope of our present study.

The presented data, combined with literature data, allow us to estimate the amount of ^31^P-NMR detectable bulk lipid molecules and their distribution between the different lipid phases. This can be done because, like it has been shown before, PG is homogeneously distributed in the bulk phase (Duchene and Siegenthaler 2000; van Eerden et al. 2015). Note that this does not hold true for lipid molecules that are bound to different protein complexes (Hölzl and Dörmann 2019; Yoshihara and Kobayashi 2022); these, so-called non-annular or structural lipid molecules do not contribute to the measured spectra.

It is generally agreed that the average protein coverage in granum thylakoids is cca. 70% of the surface area (Haferkamp and Kirchhoff 2008), leaving 30% for lipid occupancy. Indeed, applying image processing on Fig. 5  of (Boekema et al. 2000), i.e. the packing of PSII supercomplexes in granum membrane, we found that lipids occupy 30.4% of the total area (SFig. 6). In the following calculations, we use the approximation that a granum possesses a diameter of 500 nm (Mazur et al. 2020). Taking these values, we obtain that the total surface area of a granum membrane equals to 196,350 nm^2^; and that lipids cover an area of 59,690 nm^2^. Using the estimation that 60% of these lipids belong to the bulk lipid phase and 40% to the shell (Kirchhoff et al. 2002; Páli et al. 2003), we obtain that bulk lipids occupy 35,814 nm^2^. Using I (I_1_ + I_2_) contributions of 14.2%, we obtain an area of 5,086 nm^2^, which (if evenly distributed along the perimeter of the membrane) may form a thin (an estimated 3.3 nm wide) rim. Evidently, we cannot take the face values of these figures because all the rough estimations and the error bars involved in the geometrical and the bulk-lipid volume parameters as well as in the lipid-phase contributions. Nonetheless, the estimated values permit the formation of hemifusion channels constituted by fast-tumbling lipid molecules that are not ‘locked’ in the small bilayer patches between PSII supercomplexes (SFig. 4). High resolution structural investigations and micro-spectropolarimetry, might provide further information about the nature of the regions responsible for the fusion of BBY membrane particles.

**Fig. 2:**
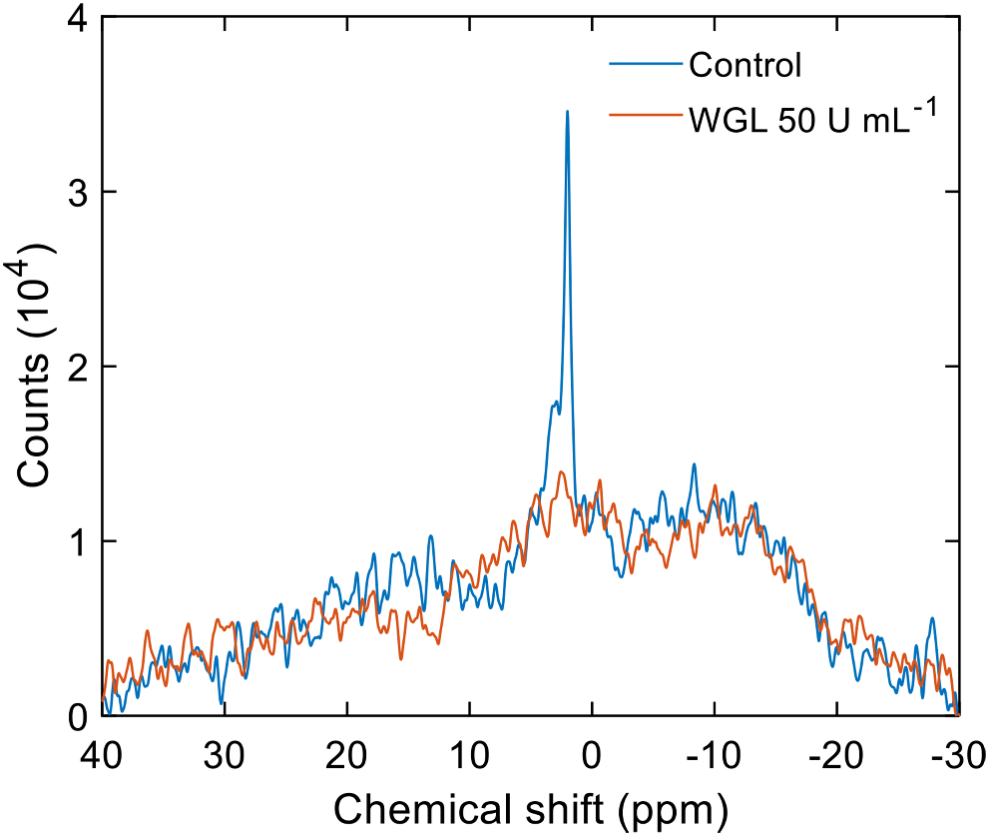
^31^P-NMR spectrum of untreated BBY particles isolated from spinach (Control, blue) and treated for 1 h with 50 U mL^-1^ WGL (orange). Spectra from three independent biological replicates were averaged. Number of scans: 6400 (per replica)

#### Selective diminishment of the I phases by WGL

WGL has earlier been shown to selectively suppress the I phases of TMs and granum and stroma subchloroplast particles in a concentration-dependent manner (Dlouhý et al. 2021a, 2022). As expected, WGL eliminated the I phases of BBY, with no discernible effect on the L phase (Fig. 2). The magnitude of this effect depended on the concentration of WGL (SFig. 7) albeit the sensitivity varied from preparation to preparation (data not shown).

**Fig. 3:**
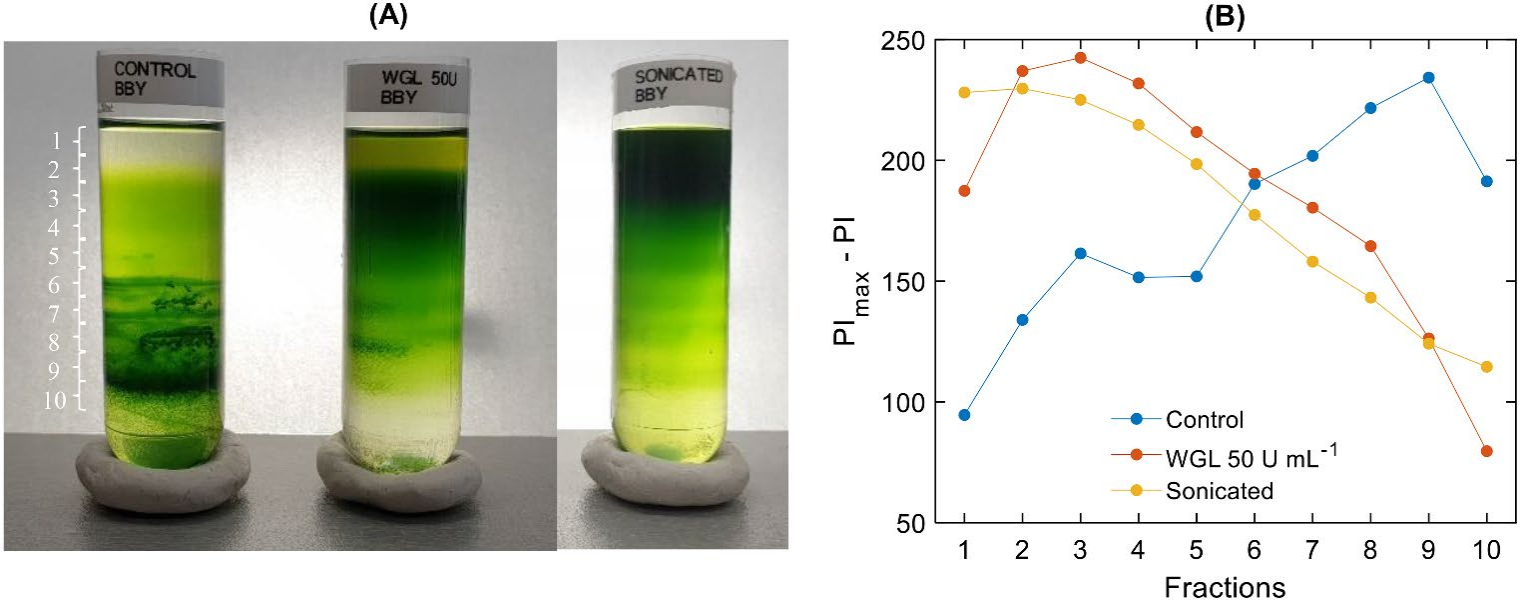
Sucrose density gradient fractionation of untreated (Control), WGL-treated (50 U mL^-1^) and sonicated BBY particles (**A**); and the corresponding pixel intensity plots (**B**) along the tube from top to bottom

In good agreement with our earlier data, obtained on TMs and granum and stroma membrane preparations, WGL exerted no effect on the molecular organization and photochemical activity of PSII supercomplexes. This was concluded from comparisons of CD spectra and fast Chl-a fluorescence transients recorded before and after WGL treatments (SFigs. 8 and 9, respectively). Further, in perfect agreement with our earlier findings on TMs (Dlouhý et al. 2022), WGL treatment of BBY membranes induced no noticeable alteration in the organization of the pigment-protein complexes, as testified by CN-PAGE and SDS-PAGE experiments (data not shown). Hence, these observations corroborated our earlier conclusion (Dlouhý et al. 2022) that the isotropic phases are located outside of the protein-rich regions, i.e. the bilayer embedding the PSII supercomplexes. These data also show that the primary target of WGL, i.e. the structural entity that is most susceptible to this lipase, is a subdomain of TMs that contain a large number of highly mobile lipid molecules (sharp I phases). This subdomain appears to be more accessible to WGL and thus more easily hydrolysed by this lipase than the lipids in the bilayer, where the steric hindrance of the pigment-protein complexes might also interfere with the lipase activity. In TMs, lipid phases outside of the regions enriched in the supercomplexes have been proposed to be involved in the junction of granum and stroma membranes and in areas where adjacent stroma lamellae are merged (Garab et al. 2022).

As pointed out in the Introduction, non-bilayer lipids with preference for non-lamellar structures with negative curvature induces and stabilizes fusion stalks. These non-bilayer phases with isotropic features (see e.g. Fig. 7F-H in (van Eerden et al. 2015)), are key components or intermediate structures in membrane fusions (Seddon and Templer 1995; Chernomordik and Kozlov 2008; Joardar et al. 2022). These data strongly suggest that I phase might be involved in the fusion of the several hundred nanometer sized PSII membranes into large sheets with up to tens of micrometer size diameters (Dunahay et al. 1984). To test this hypothesis, we investigated the effect of the WGL-induced elimination of the I phases on the size distribution of the membrane sheets.

### Disintegration of the membrane sheets by WGL

#### Size distribution of particles – SDG

Sucrose density gradient centrifugation is a technique used to separate particles based on their size, shape and density (D’Amici et al. 2009). In the case of homogeneous samples such as BBY, containing essentially only PSII supercomplexes, the distribution of particles along the gradient is determined by the size of the particles. As seen in Fig. 3A, the untreated particles tended to accumulate in the lower regions at high sucrose concentrations, indicating the presence of large membrane particles in the sample. Upon WGL treatment, 50 U mL^−1^ at 5 °C 30 min, the majority of the sample appeared on the top of the density gradient column, in regions at low sucrose concentrations (Fig. 3A), indicating the presence of smaller particle sizes. A semi-quantitative determination of the size distribution of untreated and treated samples was obtained via plotting the PI_max_ - PI values, which are characteristic of the optical density of the selected volumes, i.e. proportional to the Chl concentration. It is to be noted that without calibration this plot provides only semi-quantitative information. Nevertheless, Fig. 3B clearly shows the redistribution of BBY particles in WGL-treated sample and accumulation of smaller sized particles. Experiments using gentle sonication to disassemble BBY membranes confirmed that the WGL-induced redistribution of particles can indeed be attributed to the disintegration of large sheets into smaller units (Fig. 3A and B).

**Fig. 4:**
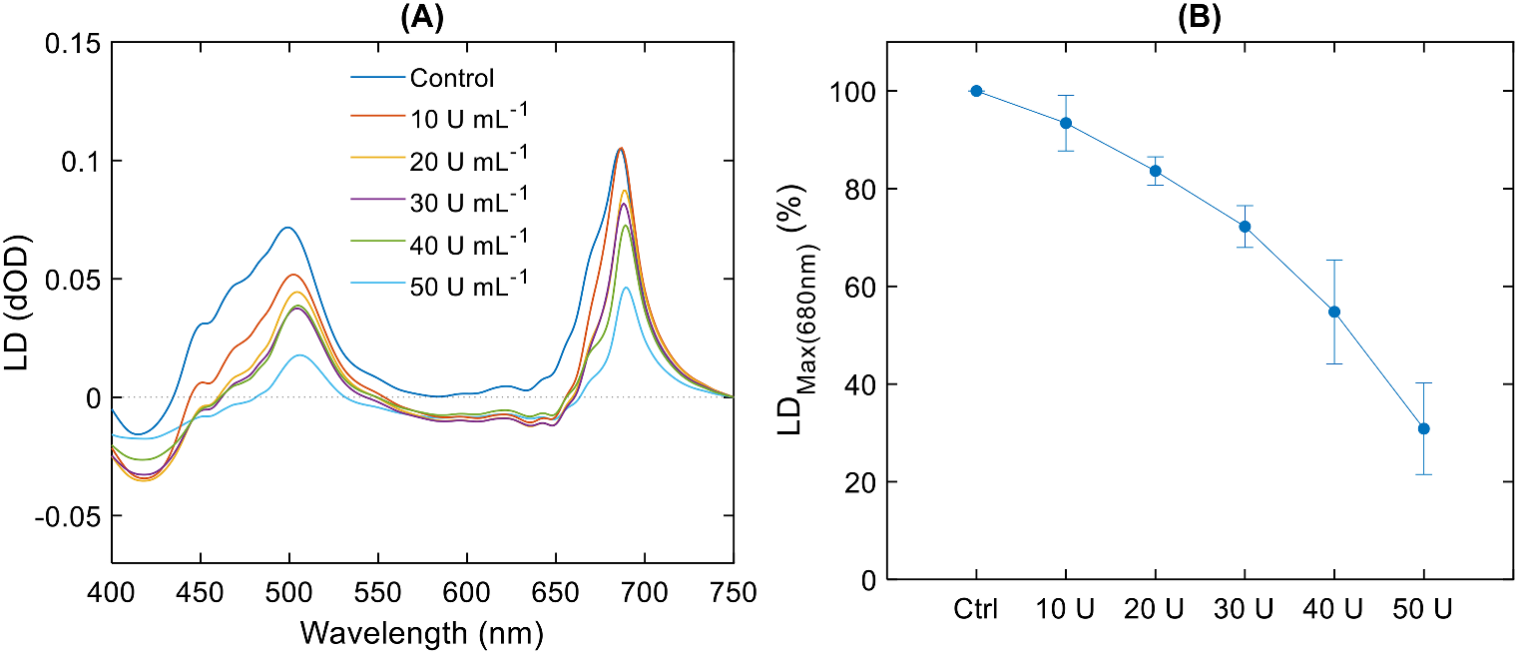
Linear dichroism spectra (**A**) of BBY membranes in the absence and presence of WGL, with gradually increasing concentration (10–50 U mL^-1^) of the lipase, and the dependence (**B**) of LD_max_ in the red spectral region on the WGL activity (*n* = 3). The maximum intensity for each untreated sample was taken as 100%; mean values ± SD obtained from three independent experiments

#### Magnetic alignability of particles – LD spectroscopy

LD measurements provide information about the anisotropy of the absorbance transition dipoles of the sample. The magnitude of LD depends on the efficiency of the alignment of membranes. In the case of magnetic orientation, at the same field strength and homogeneous sample, it depends largely on the size of the particle, determining the magnitude of the summed diamagnetic anisotropy vector of the particle (Kiss et al. 1986; Barzda et al. 1994).

When subjecting the large flat sheets of laterally fused BBY particles to a magnetic field strength of 0.7 T to induce alignment, we observed the characteristic distinct LD signal of the prominent peaks at approximately 690 nm and 500 nm (Fig. 4A). This spectrum agrees well with those in earlier reports (Tapie et al. 1982). Upon treating the membranes with different concentrations of WGL, ranging from 10 U to 50 U mL^−1^, a gradual reduction in the LD signal was observed (Fig. 4B). This can evidently be attributed to the gradual diminishment of the particle size. The proposed mechanism is schematically illustrated in SFig. 10.

**Fig. 5:**
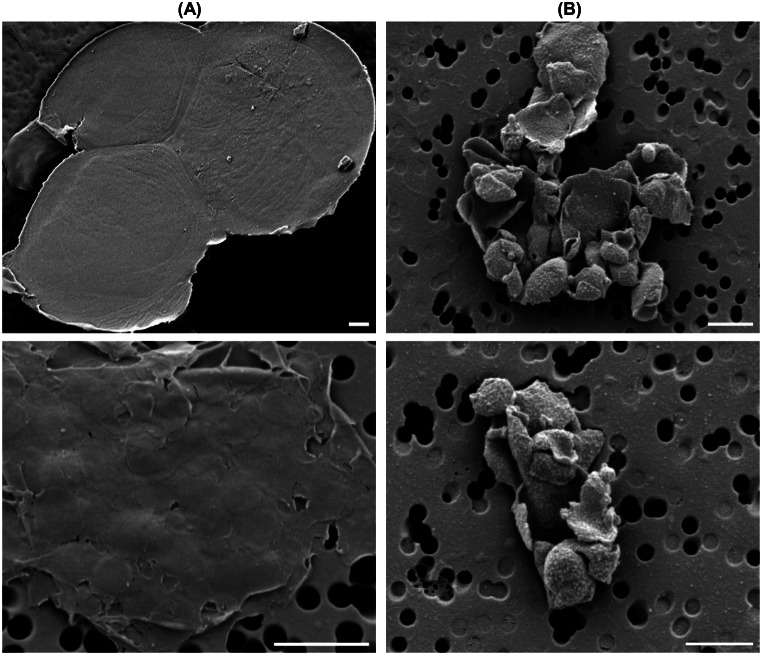
Selected images of scanning electron micrographs of control (Panel **A**) and WGL treated (Panel **B**) BBY membrane particles. Bars, 1 μm

#### Visualization of membrane particles – scanning electron microscopy

We used SEM to visualize the effect of WGL treatment on the laterally fused BBY particles. A typical pair of SEM images are displayed in Fig. 5, which confirms that BBY particles are fused together on large-sized sheets of larger than 10 μm in diameter (Fig. 5A), in perfect agreement with (Dunahay et al. 1984). As expected, WGL-treated samples contained often coagulated, smaller-sized particles, with typical diameters of several hundred nanometers, as the size of the constituent grana (Fig. 5B).

SEM experiments were also conducted to compare the mechanisms of disintegration of the large sheets of BBY by sonication and WGL treatment. As shown in SFig. 11 of the Supplementary Material, sonication disrupted the membrane sheets into small interwoven but uncharacteristic structures. In contrast, after WGL treatment, the constituent granum patches can still be recognized – corroborating the conclusion that WGL dissects BBY particles by hydrolysing the lipids located in domains interconnecting the stacked membrane pairs of grana.

## Conclusions

**Fig. 6:**
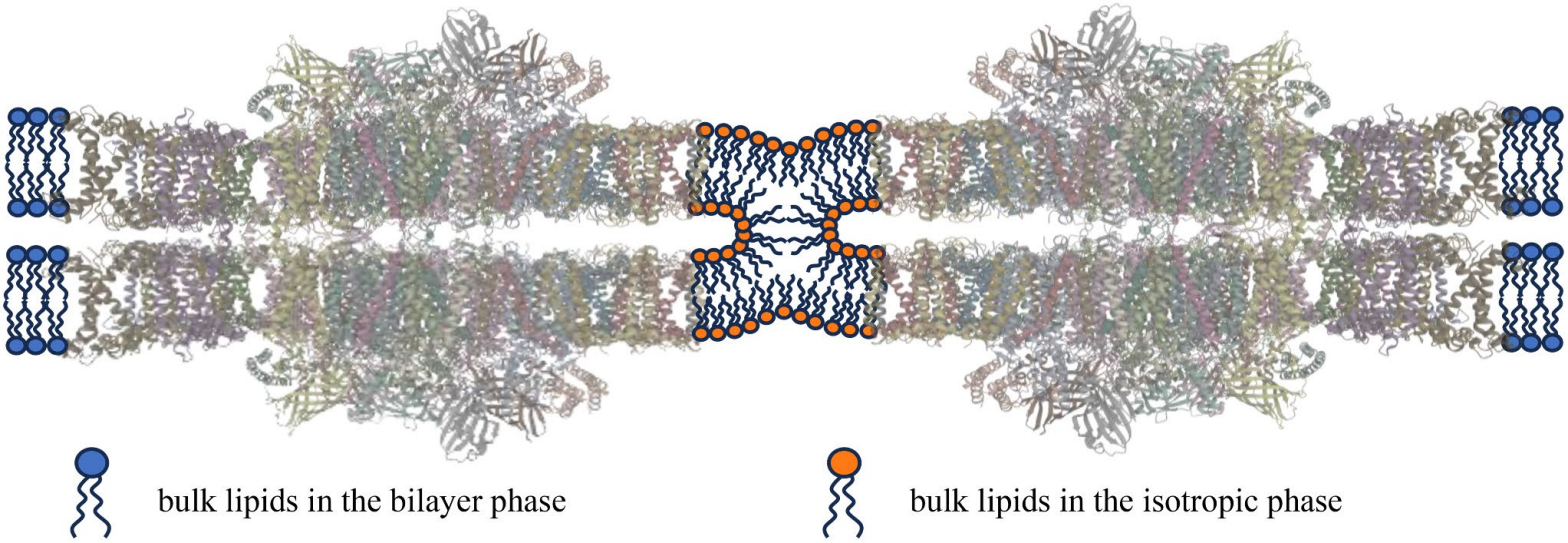
Schematic illustration of the spontaneous fusion of stacked PSII membrane pairs, BBY particles, mediated by the non-bilayer isotropic phase of TM lipids

In this paper we demonstrated the involvement of the non-bilayer, isotropic phase(s) in the fusion of PSII-enriched stacked membrane pairs, the so-called BBY particles. Typical BBY membranes are large sheets, up to tens of micrometer sizes that are composed of appressed membranes obtained from grana of several hundred nanometers in diameter. These particles exhibit characteristic lipid polymorphism: in addition to the bilayer (L phase) they contain I phases arising from rapidly moving lipid molecules, which appear to be found in regions distinct from the protein-rich bilayer phase. WGL selectively eliminated the I phase(s), which led to the marked disassembly of the extended sheets into smaller entities. It is proposed that the spontaneous fusion of BBY membranes is mediated by lipid molecules that are found on the periphery of the stacked membrane pairs. These fast-tumbling lipid molecules, assembled into I phase, might form a narrow, several nanometer wide ring around the constituent particles and ‘glue’ together adjacent membranes via forming hemifusion channels – as displayed schematically in Fig. 6.

The exact nature of the isotropic non-lamellar mesophases remains to be determined – possibly using molecular dynamic simulation techniques as well as high-resolution imaging techniques and micro-spectropolarimetry. Nevertheless, our data strongly suggest the role of non-bilayer lipids and non-lamellar lipid phases in the self-assembly and structural dynamics of TMs, in harmony with the predictions of DEM, the dynamic exchange model proposed for energy-converting membranes. The mechanism of membrane fusions outlined here appears to operate in the absence of fusogenic proteins, which however – probably in concert with non-bilayer lipid phases – may play an important role in the biogenesis of chloroplasts.

### Electronic supplementary material

Below is the link to the electronic supplementary material.

Supplementary Material 1

Supplementary Material 2

Supplementary Material 3

## Data Availability

No datasets were generated or analysed during the current study.
