# Supplementary material for: Role of isotropic lipid phase in the fusion of photosystem II membranes
Source: Photosynth Res. 2024 Apr 25;161(1-2):127–40. doi: 10.1007/s11120-024-01097-3 (PMC11269484; doi:10.1007/s11120-024-01097-3)
Supplement: Supplementary file 2 — Supplementary Material 2 [file 11120_2024_1097_MOESM2_ESM.docx]

**Role of isotropic lipid phase in the fusion of photosystem II membranes**

*Supplementary Material*


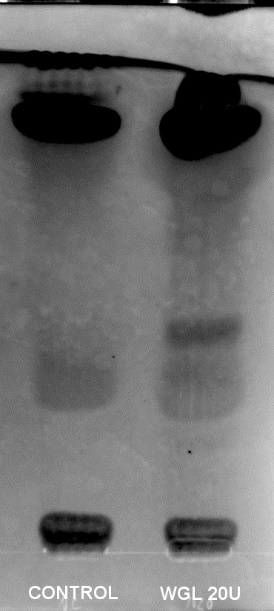


**SFig. 1**: Thin layer chromatography of MGDG (450 µg); untreated (control) and treated with 20 U mL^-1^ WGL. Samples were incubated at 4 °C for 2 h and TLC was run in chloroform:methanol:ammonium hydroxide mixture (65:25:4 v/v/v) and detected under UV light at 366 nm. Courtesy of Kristýna Večeřová (Masaryk University, Brno); this experiment was performed, but not presented, as part of the work published by Dlouhý et al. (2022).

**SFig. 2**: ^31^P-NMR spectra of freshly isolated BBY particles acquired with inter-pulse relaxation delay times (DT) of 0.5 s (red) and 2 s (black). Individual spectral components, obtained by mathematical deconvolution, of the spectra with inter-pulse times of 0.5 and 2 s are shown as brown and grey lines, respectively. Inset shows the isotropic region. Chl content, 10 mg mL^-1^, number of scans, 3200; temperature, 5°C.

| ** |  |  |
| --- | --- | --- |

**SFig. 3**: Infrared spectral features of isolated spinach thylakoid membranes and BBY particles. Panel A: Red lines indicate the 3^rd^ order polynomials fitted as backgrounds to the ‘Ester C=O + Amide I’ region. The background was subtracted from the spectra of the region of interest during its detailed analysis (see below). Panel B: averaged absorption spectra of thylakoid membranes (n = 4) and BBY particles (n = 6) of the ‘Ester C=O + Amide I’-region. The shaded areas around the average spectra indicate the estimated standard deviations. Panel C: Bar plot of the integrated area of the ‘Amide I’- region between 1700-1600 cm^-1^ and the ‘Ester’-region between 1750-1700 cm^-1^, and the protein to lipid (P/L) ratios. Note that P/L is significantly higher (13.98 ± 3.46) for BBY particles than for thylakoid membranes (9.3 ± 0.72). These data are in harmony with literature data reporting an increase in the packing density of proteins in BBY from about 70% in intact granum thylakoid membranes to about 80% in BBY particles (Haferkamp et al. 2010). Indeed, assuming 70% protein and 30% lipids in our thylakoid membrane preparations, with the mean values of the spectral data we would obtain 77.9 % in BBY.


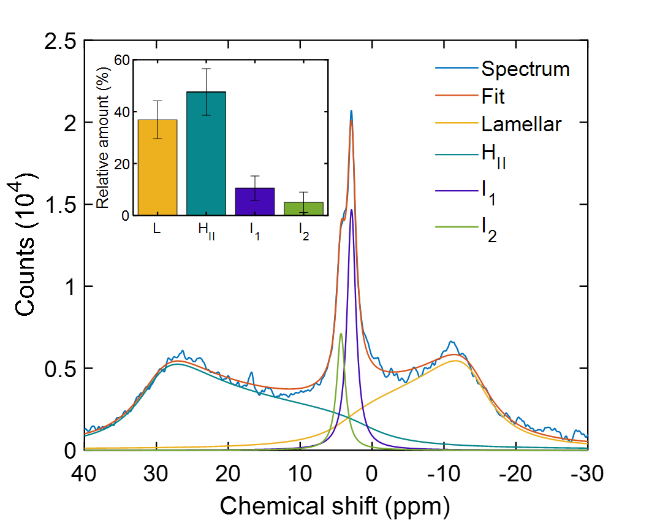


**SFig. 4:** Mathematical deconvolution of the ^31^P-NMR spectrum of TMs depicted in Fig. 1A. Relative distribution of the lipid phases: L = 36.9 ± 7.3%; H_II_ = 47.6 ± 9.0%; I_1_ = 10.5 ± 4.7%; I_2_ = 5.1 ± 3.9%. These data are very similar to those published earlier (Dlouhý et al. 2021).


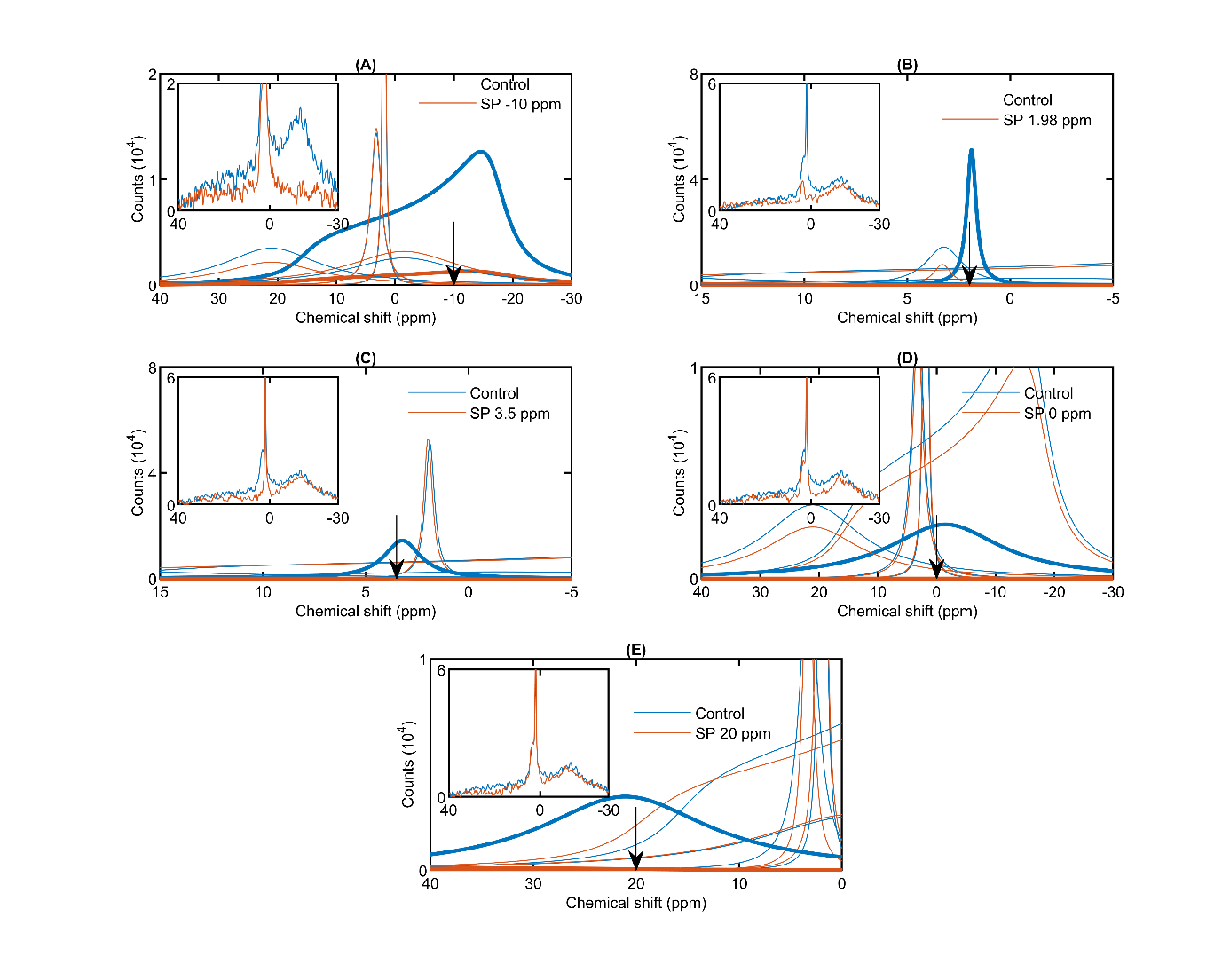
**SFig. 5:** ^31^P-NMR spectra of BBY particles in the absence (Control, blue curves) and presence (SP, red curves) of saturation pulses applied at different frequencies as indicated. The measured spectra are plotted in the insets; characteristic spectral components were obtained after mathematical deconvolution largely based on SP experiments.

**SFig.**2,. Infrared spectrum of a thylakoid membrane preparation. Red lines indicate the 3^rd^ order polynomials fitted as backgrounds to the ‘Ester C=O + Amide I’ region. The background was subtracted from the spectra of the region of interest during its detailed analysis, for details, see below. XB, Room-temperature infrared absorption spectra of thylakoid membrane and BBY particles from spinach leaves from ‘Ester C=O + Amide I’-region. The shaded areas around the average spectra indicate the estimated standard deviations within the replicates. The inset bar plot shows the L/P ratios, i.e. the ratio of signals originating from lipids (L) and proteins (P); signals which were obtained by fitting Skew-Gaussian function to the corresponding spectral regions. Note, that the L/P ratio is significantly lower (0.06 ± ?) for BBY particles as compared to that of thylakoid membranes (0.1 ± ?). **The L/P ratio should be calculated and the frequency axis should always go from 1750 to 1600** cm^‑1^ (B) An illustration of the quality of the Skew-Gaussian fit (for BBY particles). The quality is the same for thylakoid membranes (fit not shown). For calculating the L/P ratio, the lipid bands (1727,1745 cm^-1^), and the protein bands (1648,1658 cm^-1^) were added, respectively.

**SFig. 6:** Analysis to determine the areas occupied by proteins and lipids in the core of a granum membrane – the image in Panel A is reproduced (with permission from Academic Press) from the publication of Boekema et al. (2000); Panel B illustrates the lipid (white) and protein (black) covered patches, respectively; and the bar diagram (Panel C) shows the corresponding ratios of occupied areas: lipid area 30,4% and protein area 69,6%.


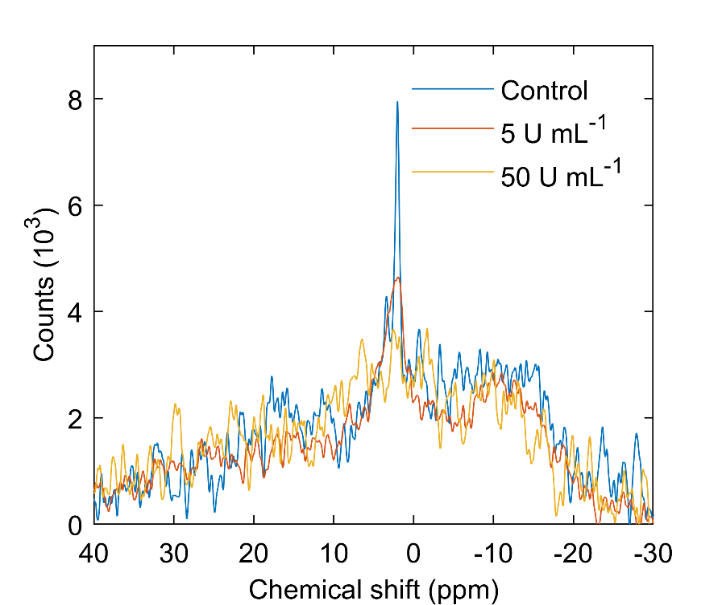


**SFig. 7:** ^31^P-NMR spectra of untreated and 5 U mL^-1^ and 50 U mL^-1^ WGL-treated BBY particles. Note that 5 U mL^-1^ WGL diminishes but does not fully eliminate the I phases.

**SFig. 8**: Circular dichroism (CD) spectra of BBY membranes in the absence (Control) and presence of 10 and 50 U mL^-1^ WGL, as indicated. Apart from the minor effects in the 410-430 nm region, WGL exerted no characteristic alteration in the excitonic band structure of the samples.

**SFig. 9**: Chl-a fluorescence transients (Panel A) and F_v_/F_m_ values (Panel B) of untreated (Control) and WGL-treated BBY particles. Effect of WGL treatment (50 U mL^-1^ for 10, 20 and 30 min) on the Chl-a fluorescence transients (A) and the calculated F_v_/F_m_ parameters (B).

*
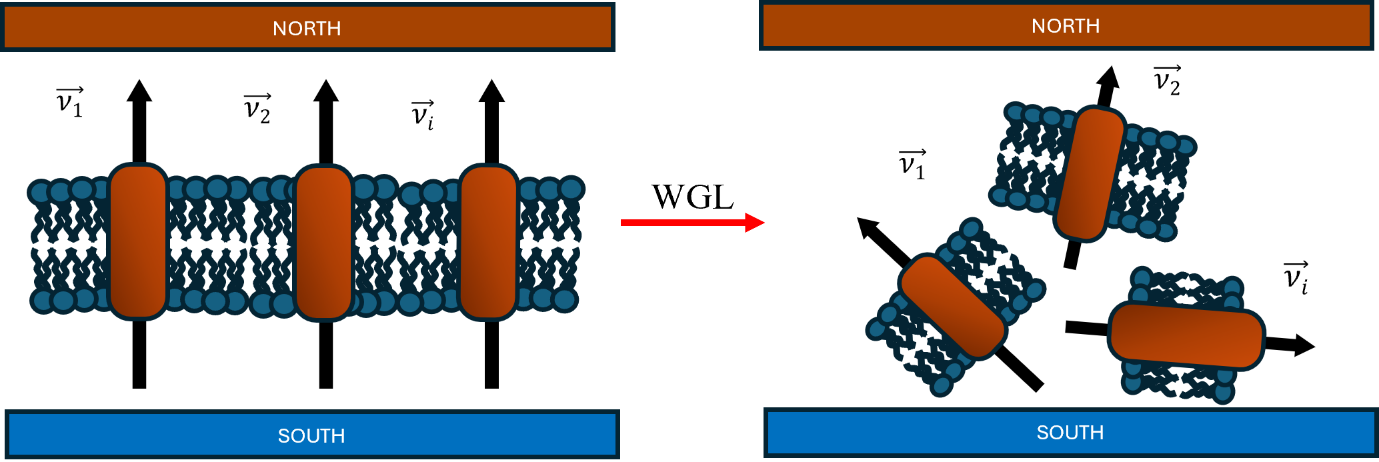
*

**SFig. 10**: Schematic figure illustrating how WGL-induced disassembly and/or disorganization of the laterally fused PSII (BBY) membranes leads to the substantial diminishment of the diamagnetic anisotropy of the sample. In large sheets the sum of the vectors is large enough to allow magnetic alignment of the sheet and thus it displays sizeable LD in an external magnetic field of 0.7 T; this does not hold true for the constituent units.

| Control | Sonicated | WGL 50 U mL^-1^ |
| --- | --- | --- |
| 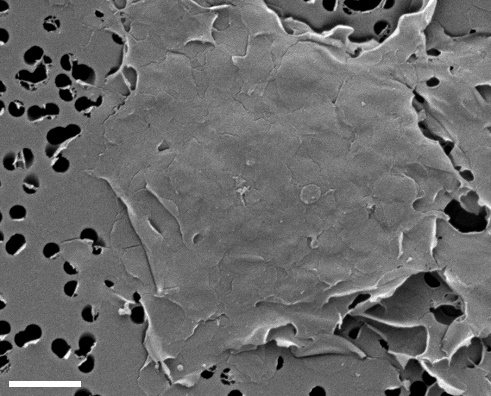 | 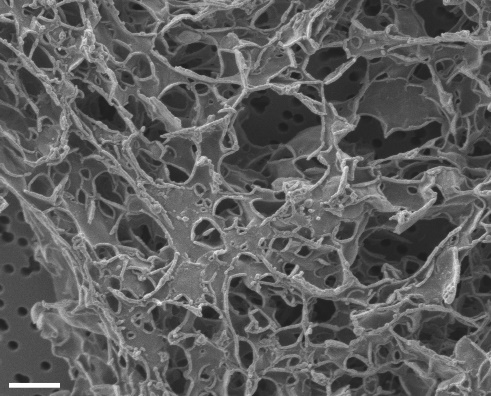 | 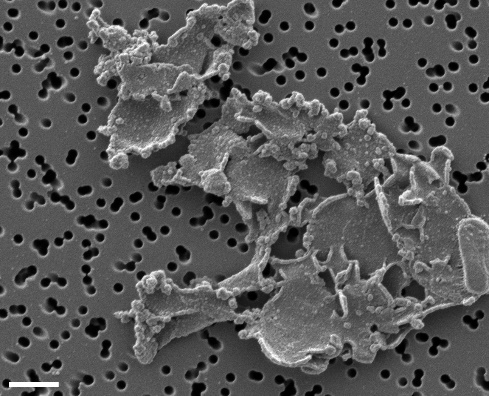 |
| 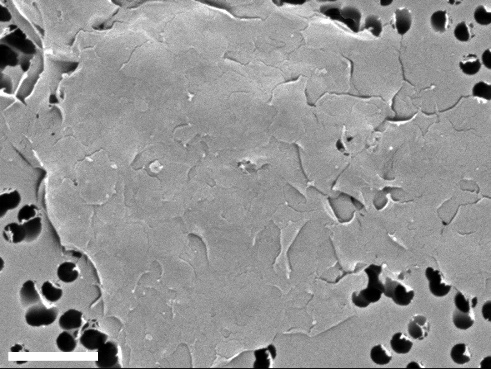 | 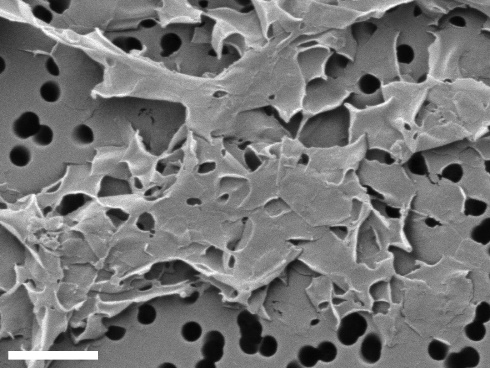 | 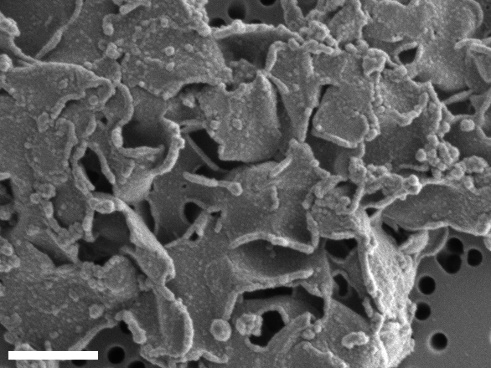 |

**SFig. 11**: Scanning electron micrographs of control, sonicated WGL-treated (50 U mL^-1^) BBY particles (left. middle and right panels, respectively). Scale bar in each micrograph, 1 µm. It is clear that both sonication and WGL treatment dismantle the large sheets of BBY membranes. It is noteworthy that sonication appears to disrupt non-selectively the membrane sheets, with WGL treatment the constituent units obtained from grana can be recognized.
